# Sub-Lethal Effects of Pesticide Residues in Brood Comb on Worker Honey Bee (*Apis mellifera*) Development and Longevity

**DOI:** 10.1371/journal.pone.0014720

**Published:** 2011-02-23

**Authors:** Judy Y. Wu, Carol M. Anelli, Walter S. Sheppard

**Affiliations:** Department of Entomology, Washington State University, Pullman, Washington, United States of America; INRA - Paris 6 - AgroParisTech, France

## Abstract

**Background:**

Numerous surveys reveal high levels of pesticide residue contamination in honey bee comb. We conducted studies to examine possible direct and indirect effects of pesticide exposure from contaminated brood comb on developing worker bees and adult worker lifespan.

**Methodology/Principal Findings:**

Worker bees were reared in brood comb containing high levels of known pesticide residues (treatment) or in relatively uncontaminated brood comb (control). Delayed development was observed in bees reared in treatment combs containing high levels of pesticides particularly in the early stages (day 4 and 8) of worker bee development. Adult longevity was reduced by 4 days in bees exposed to pesticide residues in contaminated brood comb during development. Pesticide residue migration from comb containing high pesticide residues caused contamination of control comb after multiple brood cycles and provided insight on how quickly residues move through wax. Higher brood mortality and delayed adult emergence occurred after multiple brood cycles in contaminated control combs. In contrast, survivability increased in bees reared in treatment comb after multiple brood cycles when pesticide residues had been reduced in treatment combs due to residue migration into uncontaminated control combs, supporting comb replacement efforts. Chemical analysis after the experiment confirmed the migration of pesticide residues from treatment combs into previously uncontaminated control comb.

**Conclusions/Significance:**

This study is the first to demonstrate sub-lethal effects on worker honey bees from pesticide residue exposure from contaminated brood comb. Sub-lethal effects, including delayed larval development and adult emergence or shortened adult longevity, can have indirect effects on the colony such as premature shifts in hive roles and foraging activity. In addition, longer development time for bees may provide a reproductive advantage for parasitic *Varroa destructor* mites. The impact of delayed development in bees on *Varroa* mite fecundity should be examined further.

## Introduction

Losses associated with colony collapse disorder (CCD) represent a continuation in sudden and often catastrophic population crashes in honey bee (*Apis mellifera)* colonies that have become commonplace since the mid 1980s, when two species of parasitic mites were discovered in the United States [1]. Over 60 contributing factors of CCD have been identified, including *Varroa destructor* mites, poor nutrition, exposure to both agrochemicals and beekeeper-applied pesticides, and various other pests and pathogens [2]. Honey bee health decline and colony losses have not been limited to the U.S. Many studies in Europe have examined potential correlations between major recent bee losses and pesticide exposure, particularly, the class of neonicotinoid insecticides [3], [4], [5]. Studies from Spain have focused mainly on the effects of *Nosema ceranae,* a microsporidian pathogen that targets the honey bee midgut and deprives infected bees of nutrients [6]. There is a lack of agreement about which factors are more important in colony collapse and some researchers have focused on interaction effects of combined factors. For example, pesticide exposure increases honey bee susceptibility to *Nosema ceranae* spore infection and *vice versa* [7], [8].

Honey bee colony health can be affected by many factors including hygienic behavior, innate immunity, pesticide sensitivity, nutrition, adult age, and temperature. As social insects, honey bees have evolved various traits, such as grooming or other hygienic behaviors (including removal of mites and dead or diseased brood) that protects the colony against pests and pathogens. Social immunity provides significant protection for honey bee colonies and it has been suggested that this may explain why, compared to non-social insects, honey bees are relatively immunologically deficient (i.e., express fewer immune response proteins) [9]. Honey bees have about half as many detoxifying enzymes as pesticide resistant insects [10]. This deficiency increases the sensitivity of honey bees to pesticide exposure and can further reduce their ability to fight bacterial or viral infections. Pesticide sensitivity and physiological condition may also vary due to bee age and nutritional status, which can affect overall colony health [11]. Older bees (foragers) are more susceptible to pesticide exposure due to foraging activity than younger bees that remain in the hive [12], [13]. Honey bees fed high quality pollen are less susceptible to pesticide exposure than bees fed protein-deficient pollen or pollen substitutes [12]. Migratory commercial beekeepers typically provide pollen substitute to colonies during transport and seasonal dearth to maximize brood production prior to and during pollination services. Adult honey bees are also more susceptible to pesticides when reared at lower temperatures (33°C) [14], a potential added stress factor associated with the commonly employed transportation of honey bee colonies.

In this study we examined the sub-lethal effects of developmental exposure to pesticide residues on worker bees. Worker bees were reared in brood comb containing high levels of known pesticide residues or in brood comb relatively free of pesticide residues. We discuss implications of sub-lethal and indirect effects of pesticide residues in brood comb on colony health and structure.

## Materials and Methods

### Experimental combs

Frames of treatment brood comb originated from migratory Pacific Northwest beekeeping operations that used miticides and from colonies provided by the USDA- ARS honey bee laboratory, Beltsville, MD that were suspected to have died from Colony Collapse Disorder. Pesticide residue analyses were performed on brood comb samples and thirteen frames of brood combs positive for high levels of pesticide residues were cut into treatment blocks (11×11-cm), each containing roughly 450 cells. Control brood combs were newly drawn out from a single colony or sampled from feral colonies that tested negative for pesticide residue contamination.

### Experimental design

Standard Langstroth frames, with the center area (22×11-cm) of the frame removed, were used as frame supports for a pair of comb blocks, i.e., one low pesticide residue control comb placed next to a treatment comb block containing high pesticide residue levels (n = 17). Three colonies of similar strength were used from May through August of 2008 and 2009 to host experimental frames supporting paired comb blocks. Placing control and treatment combs within the same colony during larval development equalized possible effects of colony activity and quality of resources fed to brood. Laying sister queens from each colony were caged for 24 hours over experimental frames, allowing access to both control and treatment comb blocks. Queens were released the following day and excluded to the bottom box for the duration of the experiment. Frames containing a patch of 224 eggs on control and treatment blocks were photographed and frames with insufficient number of eggs were removed from the experiment. Egg patches were monitored for larval mortality on days 4, 8,12, and 19 of development, and photographs taken of larvae developing in control and treatment comb were mapped using Microsoft Paint 2007. On day 19, experimental frames containing pupae reared in control and treatment comb were incubated at 33±1°C. Push-in cages were used to isolate treatment and control blocks. Emergence of adult bees was recorded daily and bees were counted, tagged with Testor’s enamel, and placed in a 3.2 mm mesh metal cage (11×9×5-cm). Bees reared in treatment blocks were placed in the same cage with bees reared in corresponding control blocks from the same frame. Worker bees were fed water, 50% sucrose syrup, and pollen supplement *ad libitum* and mortality was recorded daily. Some experimental frames (n = 9) containing a pair of control and treatment comb blocks were reused up to three times during the experiment. Experimental frame supports containing comb blocks that had not yet been used in the experiment (Rep 1) were introduced to host colonies at the same time as other frames that had gone through multiple brood cycles (Rep 2 & 3) to minimize seasonal foraging and thermal effects on larval survival. A total of twenty-eight replicates were completed in this study between May and August 2008 and 2009.

### Chemical analysis

Brood comb samples were sent to Roger Simonds USDA-AMS-National Science Laboratory, Gastonia, NC to be analyzed using QuEChERS method. Pesticide residue extraction and analysis was accomplished using liquid chromatography combined with tandem mass spectrometry (LC/MS/MS - Agilent 1100 LC equipped with a Thermo Quantum Discovery Max Triple Quadrupole Mass Spectrometer or equivalent), gas chromatography coupled with mass selective detection in electron impact mode (GC/MS-EI - Agilent 6890 GC equipped with a Agilent 5975 Mass Selective Detector in EI mode or equivalent), and gas chromatography coupled with mass selective detection in negative chemical ionization mode (GC/MS-NCI - Agilent 6890 GC equipped with a Agilent 5975 Mass Selective Detector in NCI mode or equivalent). Pesticide residues extracted from comb samples were quantified using matrix matched calibration standards of known concentrations prepared from neat standard reference material. Measurements were reported in nanograms of active ingredient per gram of wax (ng/g) or parts per billion (ppb). Identification of extracted residues was achieved through mass spectral comparison of ion ratios with standards, 171 of the most commonly used pesticides and their metabolites, of known identity. Limits of detection were in the low parts per billion (ppb).

### Measurements

To assess the sub-lethal effects of exposure to pesticide residues, biologically meaningful parameters were measured throughout the main stages of the honey bee life cycle. Egg eclosion, or successful hatching was measured at day 4; larval mortality and development time from egg to pupa were recorded at day 8; pupation was recorded at day 12 and 19; adult emergence rate was recorded on day 20 and continued daily until emergence was no longer observed; and adult longevity was recorded daily until all caged bees were dead. Observations of abnormal larval development and signs of disease or pest infection were also recorded. Taken together, these life cycle parameters enabled assessment of the health effects of exposure to sub-lethal pesticide residues in brood comb.

### Statistical analysis

Pairwise comparisons with repeated measures were performed on larval mortality, adult longevity, and adult emergence rate of worker bees reared in relatively uncontaminated brood comb and brood comb containing high levels of pesticide residues. Comparisons of both treatments were made by sample day (4, 8, 12 and 19) and by the number of brood cycles (Rep 1, 2, 3). Differences in pesticide analyses, specifically the number of pesticide residues and the levels detected in control and treatment comb used multiple times, were compared before and after the experiment. Normality assumptions were accepted for bee mortality on day 4, 9, 12, and 19 in both control and treatment combs (Shapiro-Wilk W = 0.844 and 0.929, respectively). Statistical differences were detected by one-way analysis of variance (ANOVA) followed by paired two-tailed t-tests on control and treatment combs with significance determined at *p*≤0.025.

## Results

### Chemical analysis of brood combs

The number of different pesticide residues detected in treatment combs ranged from 4 to 17, averaging 10. The total number of pesticides detected in all treatments was 39 including 7 fungicides, 2 herbicides, 23 insecticides (miticides included) and 7 metabolites (Table 1). The three most frequently detected pesticide residues in treatment combs were the beekeeper applied miticides fluvalinate, coumaphos, and coumaphos oxon metabolite. Fluvalinate, a pyrethroid pesticide, was detected in treatment combs at levels as high as 24,340 ppb and averaged 6,712 ppb. Coumaphos and its oxon metabolite were detected at levels as high as 22,100 ppb and 3,140 ppb, averaging 8,079 ppb and 596 ppb, respectively. Coumaphos was the only residue detected in newly drawn out control combs (21 ppb).

**Table 1:** Pesticide residues detected in treatment combs (n = 13) used to rear worker bees in experiments.

Active ingredient	Chemical Family	Purpose of use	Toxicity honey bee	Average (ng/g)	% detected	min	max	LOD
2,4 Dimethylphenyl formamide (DMPF)		metabolite		145	15	142	147	4
3-hydroxycarbofuran		metabolite		23	8	*	23	4
Aldicarb	Carbamate	INSECT	High	20	8	*	20	4
Azoxystrobin	Strobilurin	FUNG		19	38	5	29	2
Boscalid	Carboxamide	FUNG		35	15	35	64	4
Carbendazim (MBC)		metabolite		21	31	4	48	5
Carbofuran	Carbamate	INSECT	High	32	8	*	32	5
Chlorothalonil	Chloronitrile	FUNG		17	62	4	66	1
Chlorpyrifos	Ogranophosphate	INSECT	High	8	62	3	15	1
Clothianidin	Neonicotinoid	INSECT	High	35	8	*	35	20
Coumaphos	Ogranophosphate	INSECT	Mod	8079	100	281	22100	1
Coumaphos oxon		metabolite		596	100	10	3140	1
Cyfluthrin	Pyrethroid	INSECT	Low	43	17	8	79	2
Cypermethrin	Pyrethroid	INSECT	High	2	8	*	2	2
Cyprodinil	Anilinopyrimidine	FUNG		27	31	13	61	16
Diazinon	Ogranophosphate	INSECT	High	1	15	1	2	1
Dicofol	Organochlorine	INSECT	Low	6	23	4	8	1
Dinotefuran	Neonicotinoid	INSECT	High	97	8	*	97	30
Diphenylamine	Amine	INSECT		151	23	20	281	1
Endosulfan I	Organochlorine	INSECT	Mod	2	54	1	4	1
Endosulfan II	Organochlorine	INSECT	Mod	2	38	1	5	1
Endosulfan sulfate		metabolite		1	31	1	2	1
Esfenvalerate	Pyrethroid	INSECT	High	5	46	1	12	1
Fenhexamid	Hydroxyanilide	FUNG		46	8	*	46	6
Fenpropathrin	Pyrethroid	INSECT	High	7	8	*	7	1
Fluvalinate	Pyrethroid	INSECT	High	6712	100	164	24340	1
Imidacloprid	Neonicotinoid	INSECT	High	45	8	*	45	20
Iprodione	Dicarboximde	FUNG		283	8	*	283	20
Malathion oxon		metabolite		22	8	*	22	4
Norflurazon	Fluorinated pyridazinone	HERB		5	8	*	5	6
Oxamyl	Carbamate	INSECT	High	22	8	*	22	5
Oxyfluorfen	Diphenyl ether	HERB		2	23	1	2	1
Permethrin total	Pyrethroid	INSECT	High	103	8	*	103	10
Phosalone	Ogranophosphate	INSECT	Mod	32	8	*	32	10
Pyrethrins	Pyrethroid	INSECT	High	229	8	*	229	50
Thiacloprid	Neonicotinoid	INSECT	Low	113	8	*	113	8
Thiamethoxam	Neonicotinoid	INSECT	High	38	8	*	38	20
THPI		metabolite		96	15	93	99	50
Vinclozolin	Dicarboximde	FUNG		1	8	*	1	1

Toxicity category for honey bee: High; LD50 ≤2 µg/bee = highly toxic; Mod; LD50 2–11 µg/bee = moderately toxic; minimum and maximum ranges of pesticides detected, LOD; limit of detection.

### Brood effects

**Figure 1:**
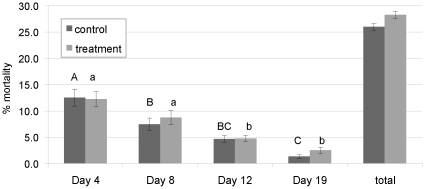
Percent larval mortality for bees reared in control and treatment comb at each sample date and overall total. Significance denoted with different letters.

**Figure 2:**
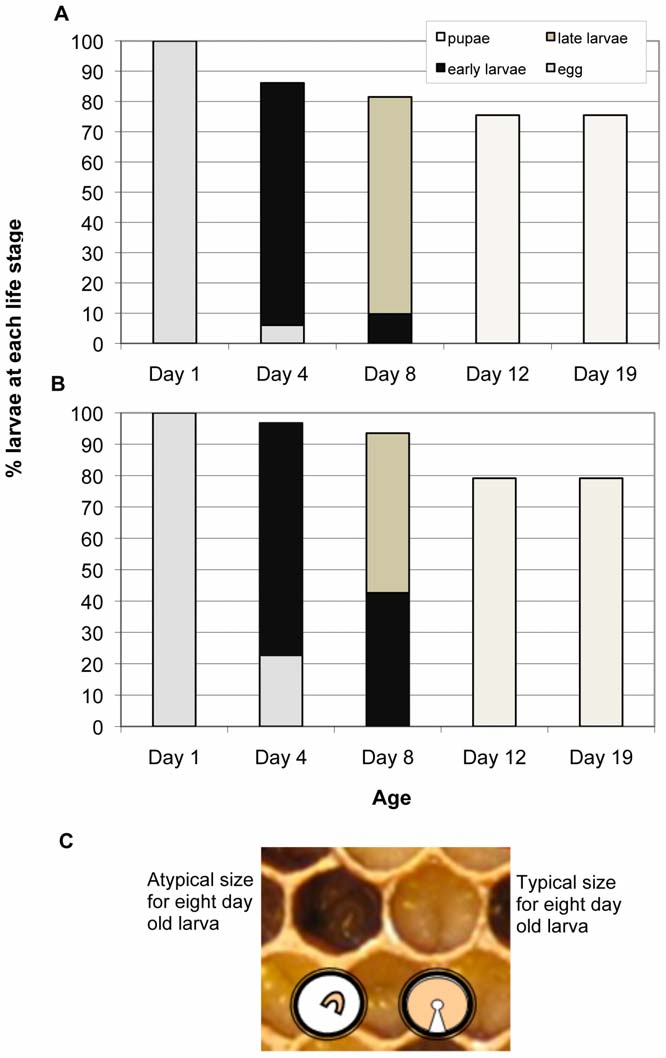
Larval development of worker bees from day 1 (egg stage) through day 19 (late pupal stage). (A) Normal larval development of bees reared in relatively uncontaminated control brood comb. (B) Larval development of bees reared in brood comb containing 17 different pesticides, expressing delayed development at day 4 and day 8. (C) Worker brood reared in brood comb containing 17 different pesticides at day 8 of development. Left: delayed growth. Right: normal development.

There was no statistical difference in total larval mortality between bees reared in control and treatment combs (26 and 33%, respectively; p = 0.059) (Fig. 1). Delayed development at day 4 and 8 was observed in bees reared from four different combs with high levels of pesticide residues originating from colonies suspected to have CCD (Fig. 2A–C).

**Figure 3:**
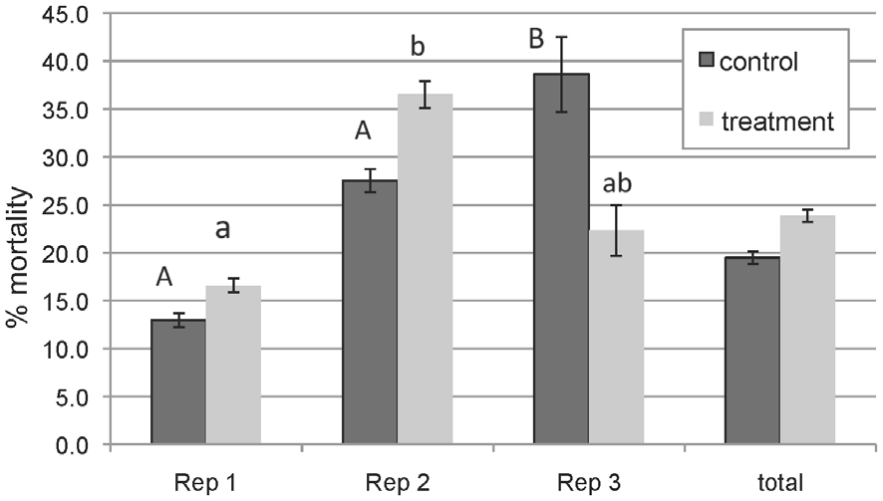
Percent mortality in larvae reared in control and treatment comb over multiple replications (Rep 1, 2, and 3; n = 28). Significance denoted with different letters.

Brood mortality in bees reared from control comb was significantly greater on day 4 of development than on days 8, 12, and 19 (p = 0.0243; p = 0.0005; p<0.0001, respectively). In contrast, brood mortality in bees reared in treatment combs was not significantly different between days 4 and 8, although mortality was significantly higher on days 4 and 8 than on days 12 and 19 (p≤0.017 and p = 0.0001, respectively). The repeated use of experimental frames over several replicates may have allowed the migration of pesticide residues from treatment to control blocks, reducing the difference in residue levels between treatment and control combs and treatment effect differences (Table 2). Mortality was significantly higher in control bees reared from frames that were used in the experiment more than once and had experienced multiple brood cycles (Fig. 3). Total larval mortality increased with the repeated use of experimental frames in control combs from 13% through the first brood cycle (Rep 1) to 28% and 39% through the second (Rep 2) and third (Rep 3) brood cycles, respectively (Fig. 3). Brood mortality in bees reared through the third brood cycle in control comb was significantly higher than in the first and second brood cycles (p = 0.023; p = 0.048, respectively). In treatment comb blocks containing high levels of pesticide residues, overall mean larval mortality increased from 17% to 37% then decreased to 22% for the first, second, and third brood cycles, respectively (Fig. 3). Brood mortality in treatment combs was significant only between the first and second brood cycles (p = 0.013).

**Table 2:** Total amount of pesticide residues detected in five pairs of control and treatment combs before & after experiments.

			Frame 1				Frame 2				Frame 3				Frame 4				Frame 5			
			Control		Treatment		Control		Treatment		Control		Treatment		Control		Treatment		Control		Treatment	
Chemical	Class		before	*After*	before	*after*	before	*after*	before	*after*	before	*after*	before	*after*	before	*after*	before	*after*	before	*after*	before	*after*
Boscalid	Carboxamide	F											35									
Chlorothalonil	Chloronitrile	F			4				66							*2*						
Cyprodinil	Anilinopyrimidine	F											61	*72*				*58*				
Fenhexamid	Hydroxyanilide	F											46									
Iprodione	Dicarboximide	F												*432*			283	*1030*				*463*
Myclobutanil	Dithiocarbamate	F																				*31*
THPI	Phthalimide	F															93				99	
Oxyfluorfen	Diphenyl ether	H											1				2					
Diphenylamine	Amine	I			281																	
Aldicarb	Carbamate	I			20																	
Carbofuran	Carbamate	I			32																	
Oxamyl	Carbamate	I			22																	
Clothianidin	Neonicotinoid	I			35																	
Dinotefuran	Neonicotinoid	I			97																	
Imidacloprid	Neonicotinoid	I			45																	
Thiacloprid	Neonicotinoid	I			113																	
Thiamethoxam	Neonicotinoid	I			38																	
Endosulfan 1	Organochlorine	I			1								1								2	
Endosulfan II	Organochlorine	I																			2	
Endosulfan sulfate	Organochlorine	I		*3*																		
Chlorpyrifos	Organophosphate	I											8	*9*			5				9	*13*
Coumaphos	Organophosphate	I		*703*	22100	*9920*	21	*4550*	281	*859*		*451*	3140	*1580*	21	*2830*	8200	*14300*	21	*669*	7230	*7090*
Phosalone	Organophosphate	I																			32	
Cyfluthrin	Pyrethroid	I			79																	
Esfenvalerate	Pyrethroid	I																			12	*6*
Fluvalinate	Pyrethroid	I		*43*	164	*159*		*1400*	11280	*2330*		*998*	24340	*14500*		*1420*	9850	*7130*		*2250*	6800	*3980*
Permethrin total	Pyrethroid	I							103													
Pyrethrins	Pyrethroid	I							229													
Paradichlorobenzene	Halogenated organic	I		*104*		*104*		*310*				*54*		*109*		*188*		*62*		*184*		*174*
2,4 Dimethylphenyl formamide (DMPF)	Amidine	m							147					*39*							142	
3-hydroxycarbofuran	Carbamate	m			23																	
Chlorferone	Organophosphate	m				*944*		*511*						*602*		*255*		*2160*				*785*
Coumaphos oxon	Organophosphate	m		*99*	1850	*617*		*276*	10	*101*		*64*	3140	*112*		*335*	474	*438*		*92*	231	*246*
Malathion oxon	Organophosphate	m			22																	
	**Total # compounds**		**0**	***5***	**17**	***5***	**1**	***5***	**7**	***3***	**0**	***4***	**9**	***9***	**1**	***6***	**7**	***7***	**1**	***4***	**10**	***9***

Results reported in ng/g or parts per billion. (F = fungicide; H =  herbicide; I = insecticide; m = metabolite).

### Chemical analysis of comb

**Figure 4:**
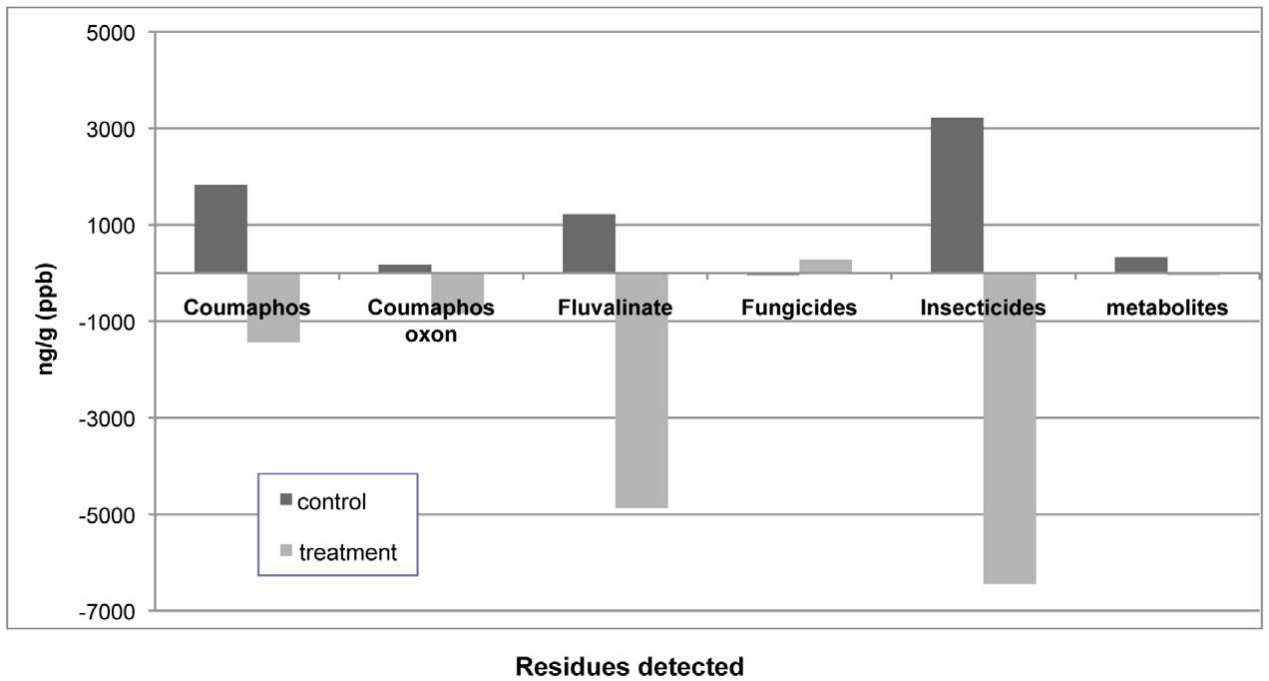
Average difference in quantity (ppb) of pesticide residues detected between pre- and post-experimental analyses for control and treatment brood combs after 2 or 3 replicates.

Comparisons of chemical analyses, performed on five paired control and treatment combs before and after the experiment (n = 10), confirmed pesticide residue transfer and contamination of control combs over a 3-month period. Four additional new pesticide residues were detected in control comb, on average, compared to a reduction of 3 pesticide residues in treatment combs after the experiment (Fig. 4). The quantity or concentration of active ingredients also increased in control combs and decreased in treatment combs after the experiment, further supporting the transfer of pesticide residue from areas of comb contaminated with high levels of pesticide residues to uncontaminated areas with low levels of residues. Insecticides, including the 3 most frequently detected compounds (coumaphos, coumaphos oxon, and fluvalinate) initially in treatment combs, increased in concentration in control combs and decreased in treatment combs after the experiment. Concentrations for coumaphos oxon, fluvalinate and combined insecticides were significantly higher in control comb after the experiment than before (p<0.025; p<0.01; p<0.025; respectively). High levels of metabolites were also detected in control combs after the experiment suggesting possible metabolism of active compounds as a result of pesticide residue migration. Fluvalinate residue levels were significantly lower in treatment combs after the experiment than before (p<0.025). The majority of new compounds found in control combs after the experiments were compounds previously detected in treatment combs at higher levels before the experiment than after (Table 2). Fungicides were the only pesticide group that was detected at higher concentrations in treatment combs after the experiment than before the experiment, an increase that was not statistically significant (averaging 280 ppb). These results illustrate that pesticide residues quickly diffuse through wax or across comb surface in an active honey bee colony.

### Adult emergence and longevity

**Figure 5:**
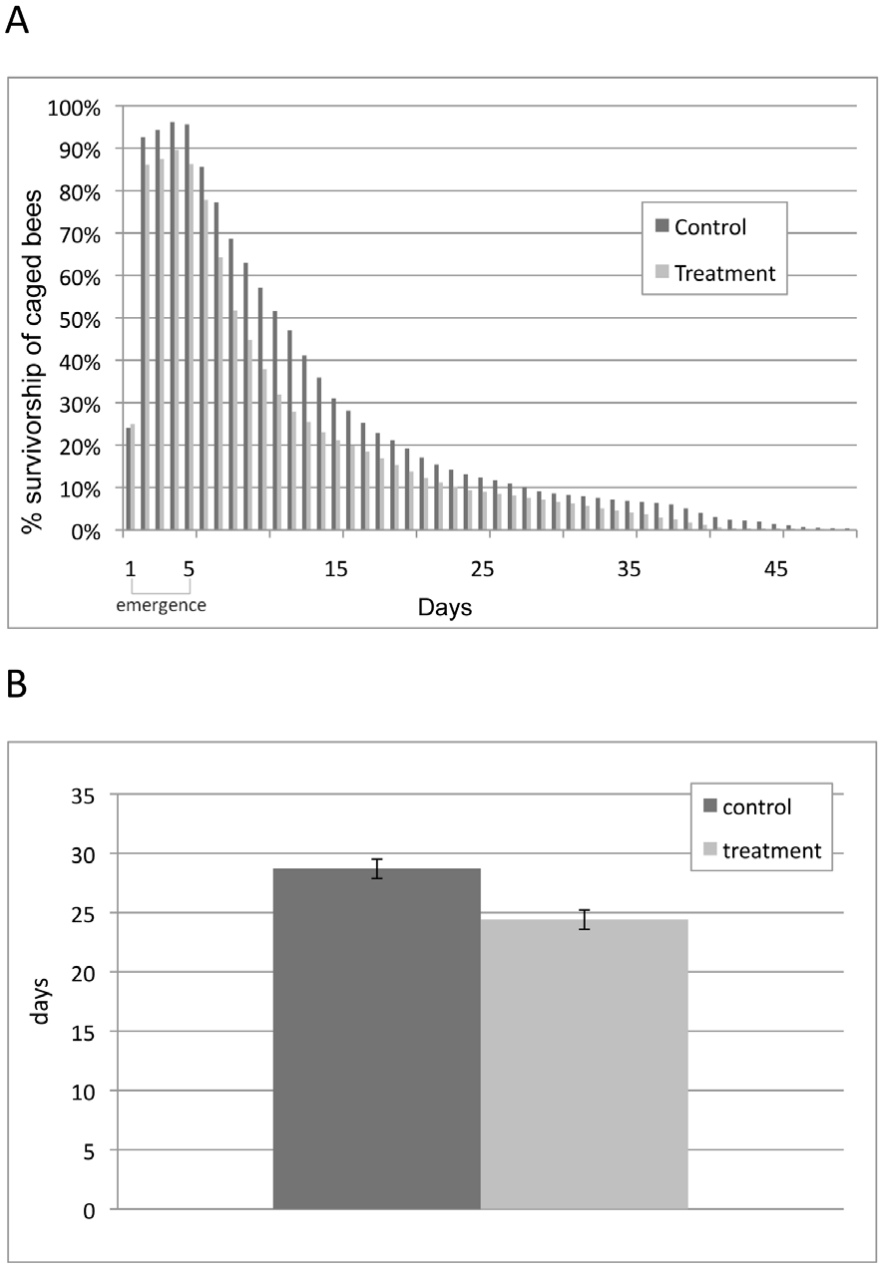
Adult emergence and longevity of bees reared in control and treatment brood comb over first brood cycle. (A) Percent emergence and survivorship of caged control and treatment bees over 50 days. (B) Average adult longevity of caged control and treatment bees. Adult bees reared in control combs lived an average of 4 days longer than adult bees reared in combs containing high levels of pesticides (p = 0.005).

**Figure 6:**
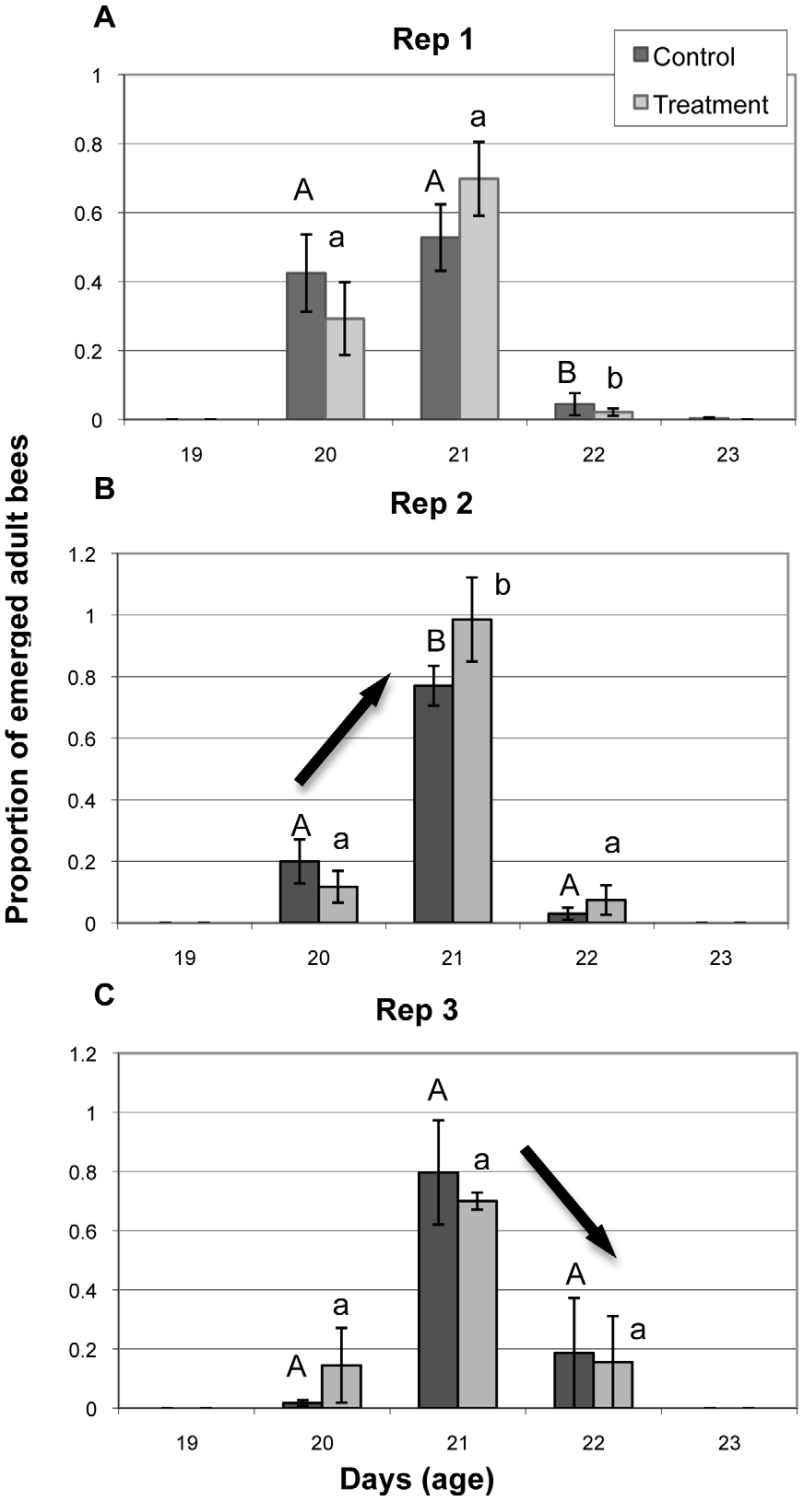
Adult emergence of bees reared in control and treatment brood comb over multiple replications. (A) First brood cycle emergence (Rep 1). (B) Second brood cycle emergence (Rep 2). (C) Third brood cycle emergence (Rep 3). Different capital letters denote significant differences in emergence of control bees on different days; different lower case letters denote significant differences in emergence of treatment bees on different days.

Worker bees reared in relatively uncontaminated brood comb lived an average of 4 days longer than bees reared in comb containing high levels of pesticide residues (Fig. 5, p = 0.005). Emergence time was also affected by contamination of control comb after multiple brood cycles, resulting in a shift in the proportion of worker bees that emerged on days 20, 21 and 22 (Fig. 6A–C). During the first brood cycle (Rep 1), a significantly higher proportion of bees emerged from control combs on days 20 and 21 of development compared to emergence on day 22. Of worker brood reared in control combs, 42% and 53% emerged as adults on days 20 and 21, respectively, while only 5% emerged on day 22 (p<0.0007). In contrast, by the third brood cycle (Rep 3) adult emergence from control comb on day 22 was much higher (18%) than emergence on day 22 during the first brood cycle (Rep 1) (5%). In addition, only 2% of worker brood reared in control comb on the third replicate emerged as adults on day 20 compared to 42% of brood that emerged on day 20 during the first brood cycle. The majority (80%) of brood from replicate 3 emerged on day 21 of development (Fig. 6A–C). These data suggest a shift in the occurrence of adult emergence from day 20–21 to 21–22 and delayed emergence for developing worker bees as a result of pesticide residue exposure to contaminated brood comb.

## Discussion

Honey bees of all ages and castes are susceptible to effects from pesticide exposure [13]. Older adult bees may be exposed to pesticides during flight and foraging, while younger adults remain in the hive but may be exposed to incoming contaminated pollen and nectar. They may also be exposed to beekeeper-applied pesticides commonly used in-hive to control *Varroa destructor* mites, serious external parasites that infect honey bee brood. Prior to adult emergence, eggs and developing bees may be exposed to pesticide residues through contaminated comb cell walls or food sources. Queen bees can be exposed to pesticides by contact with contaminated bees, wax, and food. Sub-lethal pesticide exposure through wax can have adverse reproductive consequences such as reduced egg laying, early supercedure, increased queen cell rejection, and reduced ovarian weight in queen bees [15], [16].

In this study, worker bees reared in comb containing high levels of pesticide residues had lower survivorship than bees reared in relatively uncontaminated comb. Comb age may have been a factor as well, given that brood mortality was higher in newly drawn control comb than in older control comb sampled from feral colonies. Newly drawn comb lacks exuviae (molted larval cuticles), which contain brood pheromone cues that indicate brood presence to nurse bees and increase larval survivorship [14]. However, while initial larval survivability can be lower in bees reared in new comb, overall colony health in hives using old brood comb is compromised by higher incidences of pests and pathogens [17]. The paired-block setup allowed pests or pathogens from older treatment combs to migrate or transfer (via nurse bees) over to larvae reared in new control comb. While this design was intended to help reduce differences due to pathogen loads, we cannot exclude the possibility that some pathogens, exuviae and brood pheromones embedded in the cell walls of the comb would not have been transferrable.

For economic reasons, beekeepers typically reuse wax foundation, but pesticide residues accumulate in wax and may persist for years [18]–[20]. Contamination of reused control brood combs in this experiment illustrated how quickly pesticide residues could penetrate and migrate through or across brood comb wax. The presence of additional pesticide residues in control combs detected after the experiment confirmed pesticide residue transfer and contamination of control combs. Incoming pesticides brought back by foragers from external sources would have been detected in both control and treatment combs, because the experimental frames were housed in the same colony. High levels of pesticide metabolites detected in control combs after the experiment also suggest possible metabolism of active compounds during migration. Metabolites can be more harmful to organisms than parent compounds and can have delayed effects [11], [21]. In the paired comb blocks, detection of increasing mortality for bees reared in control blocks and decreasing mortality for treatment blocks, over time, confirms toxicological consequences from pesticide residue migration.

### Brood effects of pesticide exposure

Sub-lethal effects of pesticides on bees, including delayed adult emergence, may seem inconsequential but may provide a reproductive advantage for *Varroa* mites. A gravid foundress mite can invade a cell occupied by a developing bee larva and lay four eggs in 30 hour intervals. The first egg results in a male, with subsequent eggs developing into multiple daughter mites [22]–[24]. The most injurious effects of *Varroa* mites occur when the foundress and her multiple offspring feed on the hemolymph of a pupating bee, causing reductions in emergence weight and metabolic reserves and physical deformities in host bees [25], [26]. Normally, the third daughter mite only has a 13% chance of reaching maturity before the pupating bee emerges from the cell after 20 to 21 days of development [27]. However, with delayed adult bee emergence the likelihood that the third daughter mite will successfully reach maturity and mate increases. In this study, delayed development occurred in bees reared in treatment comb containing 17 different pesticides, including 9 systemic compounds and 5 neonicotinoid insecticides (Table 3). As the queen in these experiments laid eggs in both control and treatment comb within a 24 hour period, the normal growth pattern was expected to be uniform. However, by day 4, 23% of eggs were unhatched in the treatment comb and by day 8, over 46% of remaining larvae reared in the contaminated treatment comb were small and their development visually stunted or delayed (Figs. 6A–C). Another three treatment combs, sampled from colonies suspected to have colony collapse disorder (CCD), had similar patterns of egg hatch and development. An average of 19% of eggs laid in comb sampled from CCD colonies containing high levels of pesticides remained unhatched on day 4, and 60%–90% of unhatched eggs were removed by bees before the next sampling date. Inefficiencies in brood production place energetic stresses on honey bee colonies. Nurse bees must remove unhatched eggs rather than tend to developing brood, and high brood mortality increases the demand for egg-laying by the queen. Egg-laying efficiency is further reduced when queen bees are unable to deposit eggs in a general area but, instead, must seek empty cells scattered throughout the brood nest [28].

**Table 3:** Pesticide residues contained in treatment brood comb with observed delayed development of worker honey bees.

Pesticides	Chemical family	Systemic	Toxicity honey bee	(ng/g) ppb	LOD
3-hydroxy-carbofuran	metabolite	Systemic		23	4
Aldicarb	Carbamate	Systemic	High	20	4
Carbofuran	Carbamate	Systemic	High	32	5
Chlorothalonil	Fungicide		---	4	1
Clothianidin	Neonicotinoid	Systemic	High	35	20
Coumaphos	Organophosphate		Moderate	22100	1
Coumaphos oxon	metabolite			1850	5
Cyfluthrin	Pyrethroid		High	7.9	2
Dinotefuran	Neonicotinoid	Systemic	High	97	30
Diphenylamine	Amine		---	281	1
Endosulfan 1	Organochlorine		Moderate	1	1
Fluvalinate	Pyrethroid		High	164	1
Imidacloprid	Neonicotinoid	Systemic	High	45	20
Malathion Oxon	metabolite			22	4
Oxamyl	Carbamate	Systemic	High	22	5
Thiacloprid	Neonicotinoid	Systemic	High	113	8
Thiamethoxam	Neonicotinoid	Systemic	High	38	20

Toxicity category for honey bee: High; LD50 ≤2 µg/bee = highly toxic; Mod; LD50 2–11 µg/bee = moderately toxic; LOD; limit of detection.

### Adult longevity

Worker bees reared in treatment comb containing high levels of pesticide residues lived an average of 4 days less than bees reared in relatively uncontaminated control combs in cage trials (Fig. 5). To place this in context, the mean lifespan of honey bees after entering the ranks of foragers is less than 8 days [29]. Reduced adult longevity within the ranks of foraging bees can lead to precocious foraging by “under-aged” replacement bees. Over the long term, this activity could affect an entire cascade of hive activities including brood care, food processing and storage, queen care, hygienic behavior and foraging efficiency, disrupting age-based polyethism and its role in colony homeostasis. Precocious foraging has been reported to have a major impact on colony size and viability, through reduction of the “younger” nurse bee population from which replacement foragers are derived [30]. In fact, in their model of induced precocious foraging, Thompson and co-workers [30] found that to simulate the “sublethal” effect caused by the reduction in nurse bee capabilities (the mean number of larvae that can be reared per nurse bee), the mortality rate of foragers would have to be increased by 500%.

### Conclusion

Combined effects from honey bee exposure to pesticide residue in brood comb, such as reduced adult longevity, increased brood mortality, higher fecundity of *Varroa* mites (due to delayed development and emergence of adult bees) and increased susceptibility to pathogens, may contribute to reduced honey bee colony health, as affected queens and worker bees are unable to meet the demand for brood production and resources needed to sustain large colony populations.

Honey bees are biological indicators, picking up chemicals and other pollutants from their environment both external and internal to their hives. Our findings suggest that one of the underlying commonalities in the worldwide reports of a decline in honey bee health and observations of Colony Collapse Disorder (CCD) may be exposure of honey bees and bee products to pesticides. Developmental exposure of honey bees to pesticide contaminated brood comb may appear subtle and indirect, but can lead to sub-lethal effects that actually have serious consequences.
